# The Pattern of Signatures in Gastric Cancer Prognosis

**DOI:** 10.3390/ijms19061658

**Published:** 2018-06-04

**Authors:** Julita Machlowska, Ryszard Maciejewski, Robert Sitarz

**Affiliations:** 1 Department of Human Anatomy, Medical University of Lublin, 20-090 Lublin, Poland; julita.machlowska@gmail.com (J.M.); maciejewski.r@gmail.com (R.M.); 2 Department of Surgery, St. John’s Cancer Center, 20-090 Lublin, Poland

**Keywords:** gastric cancer, HER2, cell cycle regulators, microsatellite instability, apoptosis, mucins, multidrug resistance proteins, invasiveness, peritoneal spreading, neovascularization

## Abstract

Gastric cancer is one of the most common malignancies worldwide and it is a fourth leading cause of cancer-related death. Carcinogenesis is a multistage disease process specified by the gradual procurement of mutations and epigenetic alterations in the expression of different genes, which finally lead to the occurrence of a malignancy. These genes have diversified roles regarding cancer development. Intracellular pathways are assigned to the expression of different genes, signal transduction, cell-cycle supervision, genomic stability, DNA repair, and cell-fate destination, like apoptosis, senescence. Extracellular pathways embrace tumour invasion, metastasis, angiogenesis. Altered expression patterns, leading the different clinical responses. This review highlights the list of molecular biomarkers that can be used for prognostic purposes and provide information on the likely outcome of the cancer disease in an untreated individual.

## 1. Introduction

Gastric cancer (GC) is an aggressive disease that is still a global health problem with its heterogeneous nature. In the last several decades, the decrease of gastric cancer incidence has been noted, however it is still the fourth leading cause of cancer-related death worldwide [1]. Alternative prevention, as food preservation, earlier diagnosis, and therefore, prior treatments cause abatement of recorded incidents, although the prognosis is still bad. The standard therapies include surgical resection with chemotherapy, and in suitable cases, chemoradiation [2]. To treat advanced gastric cancers and metastasis, it is still a huge barrier to be overlapped; however, little progress has been reported [3]. Gastric cancer is a heterogeneous disease; therefore, new investigation and approaches need to be considered including prevention, early detection, as well as innovative and effective therapeutic efforts.

Neoadjuvant therapy as the dosing of therapeutic agents before a major treatment of cancer, became more popular in recent studies on gastric cancer combating. The first approach in this field was reported in 1989, concerning gastric cancer patients, where cisplatin (EAP) was confirmed as an effective agent in locally advanced gastric cancer, and therefore it was a chance for surgery in patients with poor prognosis [4]. Nowadays, the importance of neoadjuvant therapy is an interesting investigation for many researchers for extension of survival time in advanced GC cases. The randomized controlled trials have been implemented; nevertheless, the final outcomes are still not consistent [5,6]. The study that was conducted by Songun et al., 1999 [7] showed that more active regimens than methotrexate (FAMTX) are required for future randomized trials.

Gastric cancer immunotherapy is another powerful approach due to better understanding of immunological networks and molecular mechanisms of immunosuppression in the cancer environment. Vaccination strategies based on protein and peptide vaccines, which can be identified by cytotoxic T and helper T lymphocytes. Currently, there are several immunotherapy-based approaches like adoptive cell therapy, where various cell types can be implemented, like lymphokine-activated killer cells [8], tumor infiltration lymphocytes (TILs) [9], and anti-CD3 monoclonal antibody-induced killer cells [10]. Dendritic cells incubated with mRNA can show the encoded antigen, which making them another vaccine type based on mRNA gene transfer [11]. Both protein and peptide targets are useful tool for stimulation of immune response, HER2/neu-derived peptide [12] and MAGE [13] which are related to MHC class I and induce cytotoxic T cells against GC cancers.

Discovering the pattern of signatures could be used as a biomarker to guide targeted therapy for gastric cancer. This heading will individualize the cancer therapy relay on integrated and global recognition of dysfunctional signalling pathways. The presented review pays attention on the currently available biomarkers for prognosis of gastric cancer. Nowadays, there are many different signatures that can provide the division of gastric carcinoma by taking in consideration age, histopathological type, *Helicobacter pylori* infection, microsatellite instability, module of HER2 expression, molecular markers of cell cycle regulators, factors that regulate apoptosis, multidrug resistance proteins, agents that influence cell membrane properties, and agents with an impact on the progression of gastric cancer and peritoneal metastasis. Radical patient selection, appropriate combinations of targeted therapies, and deployment of emerging immunotherapeutic investigations will for sure hone the treatment of this global disease.

## 2. Screening for *Epstein–Barr viruses*

Infection with *Epstein-Barr virus* (*EBV*) is associated with a wide spectrum of malignant diseases, like Hodgkin and non-Hodgkin lymphomas, post-transplant lymphoproliferative disorder (PTLD), and gastric cancer [14]. It must be clarified that *EBV* infects basically all humans by the time that they reach adulthood and the viral genome is detained in a low number of B lymphocytes. To assess *EBV* as a marker for cancer development, it is strongly advised to quantify the number of infected cells and to indicate their types. Upon primary infection, it transiently runs a short lytic program and after predominantly establishes latent infection [15]. Serological tests are commonly used to confirm the primary infection and report remote infection [16]. One of the most popular serological assays is heterophile antibody test. Cancer related to *EBV* infection is correlated with high titers against Early Antigen (EA) and IgG Viral Capsid Antigen Antibody (VCA) with low *EBV* Nuclear Antigen Antibody (EBNA) titer. Unfortunately, analogous patterns are probable in autoimmune disease and this test is not decisive when the immune system is defective, for instance, acquired immunodeficiency syndrome (AIDS) or allogeneic transplant patients.

EBER in Situ Hybridization Assay is a gold standard for localization of *EBV* in biopsied tumor [17]. *EBER1* and *EBER2* are non-polyadenylated viral transcripts, which are conjointly called *Epstein–Barr* virus-encoded small RNA (*EBER*), which are expressed at a very high level in infected cells. They have been labelled as a natural marker for latent type of infection. DNA of *EBV* is evidential within malignant epithelial cells in 10% of gastric adenocarcinomas, mostly in those arising in the stump after surgical gastrectomy and in undifferentiated with abundant tumor-infiltrating lymphocytes [18]. *EBV* positive gastric cancer patients have specific genetic mutations and epigenetic patterns profile, which is assigned to the clinical phenotype of the malignancy. It was postulated that some genes, like *BcLF1*, *BHRF1*, *BARF0*, *BARF1*, *BZLF1*, *EBNA1*, *BLLF1*, and *BRLF1* are overexpressed in GC cases with positive *EBV* infection [19,20,21]. *EBV* occurs as episomes in nuclei of host cell and averagely 205 host cell genes are altered in patients with *EBV*-positive GCs, embracing *TGFBR1*, *AKT2*, *MAP3K4*, and *CCNA1* [21]. Epigenetic alterations are also connected to the *EBV* positive gastric carcinoma. Zhao et al., 2013 [21] showed that 216 genes were hypermethylated and were transcriptionally down-regulated, and 46 were demethylated and transcriptionally up-regulated in the study that was conducted on cultured *EBV* positive GC cells. The hypermethylation status of the *IHH*, *ACSS1*, *TRABD*, and *FAM3B* genes was also found among analysed tumor samples. Targeted therapies in advanced GC with *Epstein-Barr virus* positive patients, can be applied to the *PIK3CA/Akt* pathway, *JAK2* and *PD-1/PD-L1*, and *PD-L2* pathway [22].

## 3. HER2 Status in Gastric Cancer

The HER2 receptor is a member of the Epidermal Growth Factor Receptor (EGFR) family. Its activation is going through its spontaneous homo/heterodimerization with the other EGFR family receptors [23]. Many studies have been investigated on HER2 amplification and high expression in gastric cancer, applying a different method, showing the range of positive cases between 6% and 30% [24,25,26]. GC very often displays heterogeneity of the HER2 genotype and phenotype, which can have impact on testing inaccuracy [27]. The EMEA (European Medicines Agency) recommended the IHC immunohistochemistry method (IHC) as a first screening, adding the scoring criteria, with 3+ samples to be positive and 2+ only positive, when approved by Fluorescence in situ hybridization (FISH). When considering the increased frequency of overexpression/amplification of *HER2* among GC cases, preclinical and early phase clinical studies have been conducted to assess the therapeutic value for targeted approaches [28,29]. Tanner et al., 2005 [29] investigated the occurrence and the clinical importance of *HER-2/neu* amplification in gastric carcinoma. The frequency of *HER2/neu* amplification was, respectively, 12.2% of the gastric and 24.0% of the gastroesophageal adenocarcinomas. *HER-2/neu* amplification was higher in the intestinal histologic type of gastric cancer (21.5%) in comparison to the diffuse (2%), it was not associated with age and gender, but with the poor survival of GC patients. Trastuzumab antibody that was targeting HER-2/neu inhibited the growth of a p185 (HER-2/neu) overexpressing gastric. Accuracy of trastuzumab was tested as a single dose or in combination with other chemotherapeutic agents in human gastric cancer xenograft models with high HER2 expression [28]. Trastuzumab, while added as a single drug, inhibited the tumor expansion in HER2-overexpressing models, but not in the HER2-negative. Trastuzumab with combinations of any other agents, like docetaxel, paclitaxel, capecitabine, irinotecan, and cisplatin, displayed higher anticancer action than being administrated separately. Other molecular agents targeting HER2 have been analyzed, such as pertuzumab, lapatinib, and the antibody-drug conjugate trastuzumab-emtansine (TDM-1) [28,30,31]. Unfortunately, the final effect of them has been displayed to be poor in comparison to trastuzumab, which is the first agent of targeted therapy confirmed as a standard treatment in gastric cancer.

## 4. Gastric Cancers with Microsatellite Instability

Microsatellite instability (MSI) is an important indication of the DNA mismatch repair deficiency, and therefore it is perceived as a factor in the expeditive collection of genetic changes in the gastric carcinogenesis process [32]. Hang et al., 2018 [33] presented the molecular mechanisms of of microsatellite instability (MSI) and its importance in GC prognosis. Next generation sequencing data of the whole transcriptome were investigated from The Cancer Genome Atlas (TCGA), covering 64 high-level MSI (MSI-H) gastric cancer samples, 44 low-level MSI (MSI-L) and 187 stable microsatellite (MSI-S) gastric cancer samples [33]. The results showed that MSI status might have impact on the prognosis of GC patients, partially through the inflammatory bowel disease, antigen processing and presentation, measles, toxoplasmosis, and herpes simplex infection pathways. *HLA-DRA*, *HLA-DRB5*, *JAK2*, *HLA-DQA1*, *HLA-DMA*, *CASP8*, and *Fas* could be predictive factors for gastric cancer prognosis.

Although MSI cases in general are deprived of targeted alterations, mutations in *PIK3CA*, *EGFR*, *ERBB3*, and *ERBB2* were detected [22]. Additionally, MSI tumors alteration status of genes showed common mutations in major histocompatibility complex class I genes, including *B2M* and *HLA-B.* To reveal a precise association between human mutL homolog 1 (*hMLH1*) promoter methylation and gastric cancer, a meta-analysis study was investigated by Ye et al., 2018 [34]. It was postulated that *hMLH1* promoter methylation had significant correlation with not only microsatellite instability, but also with lymph node metastasis and the low expression of hMLH1 protein. Polom K et al., 2018 [35] investigated the analysis of MSI status among cases with marginal involvement after gastrectomy, and the establishment of an association between MSI, margin status, and survival rate They displayed that patients with MSI-H gastric cancer might possess long-term survival despite positive resection margin (RM+) status. This might be important step towards surgical treatments that are based on to clinical and pathological factors [35].

## 5. CDX2 Expression in the Intestinal Type of Gastric Epithelial Dysplasia and Its Correlation with CD10

*CDX2* is a Drosophila caudal-related homeobox transcription factor, and it is a member of the caudal-related homeobox gene family. It is perceived as an important factor in mammalian early intestinal development and the regulation of intestine-characteristic gene transcription [36]. The role of *CDX2* in gastric cancer has been reported as responsible for the proliferation and differentiation of intestinal type of epithelial cells by controlling transcriptional activation of intestine specific proteins, like sucrase-isomaltase, carbonic anhydrase I, or Mucin 2 (MUC2) [37]. It also acts through the inhibition of growth by the activation of WAF1 (cyclin-dependent kinase inhibitor) [38]. In the study that was conducted by Park et al., 2010 [39], CDX2 is expressed mostly among adenomatous-type gastric epithelial dysplasia in comparison to the hybrid or foveolar types. CDX2 expression level is the lowest in the advanced gastric cancers, and it is also very decreased in the early onset of gastric cancers, indicating the possibility to act as a tumor suppressor. The study also showed that an increasing expression of CDX2 is correlated with an increased expression of CD10, which is cell surface zinc-dependent metalloprotease, also called as endopeptidase (NEP) (EC 3.4.24.11), enkephalinase, or acute lymphoblastic leukemia antigen, which in this case is promoting intestinal metaplasia of gastric epithelium. The study that was performed by Huang et al., 2005 [40] reported that CD10 expression by stromal cells was meaningfully increased in the primary gastric carcinomas in comparison to normal mucosas. CD10 expression was detected more frequently in differentiated carcinomas and it seems to cause the promotion of invasion and lymph node metastasis processes of differentiated gastric carcinoma.

## 6. Abnormalities in Cell Cycle Regulators

Genetic alterations in several regulators of the cell cycle have a significant impact on the gastric carcinogenesis process. Cyclin D1 and retinoblastoma protein (pRb) are important factors in the progression from G1 phase to S phase, which is crucial in the cell cycle [41]. In general, cyclins are positive regulators of the cell cycle process and their overexpression is linked to uncontrolled cell growth and cancer development. The main targets of cyclin D1-Cdk complexes are the retinoblastoma family of protein Rb [42]. The study that was conducted by Arici et al., 2009 [43] intimated the higher expression of Rb and cyclin D1 among nonneoplastic mucosa comprising dysplasia, intestinal metaplasia, atrophy, and gastritis to carcinoma, which indicates that the expression of pRb and cyclin D1 can occur in early stages of gastric carcinogenesis. Cyclin D1 is expressed among genetic alterations in gastric carcinomas, including advanced gastric carcinomas and early stage, using immunohistochemistry as a good standard method for the detection of early stage gastric cancers and their differentiation from hyperplastic polyp patients [44].

Another very important cell cycle regulatory protein is p16, which is cyclin-dependent kinase inhibitor (CDKI) [45]. The *p16* gene acts as a tumor suppressor gene and negatively regulates cell growth and proliferation. The relation between the inactivation of *p16* and the evolution of gastric cancer has been reported by several study groups. Deletion of *p16* gene exon 2 is related to the carcinogenesis process and progression of gastric carcinoma, which was reported by Hayashi et al., 1997 [46]. Wu et al., 1998 [47] showed that deletion of *p16* gene was oftentimes present in intestinal type of gastric cancer in comparison to the diffuse type. Hypermethylation of the *p16* locus, which causes functional inactivation, plays a relevant role in the pathogenesis of sporadic gastric cancer [48].

Cyclin-dependent kinase inhibitor 1B, called p27^Kip1^, is referred to play a role as a cell cycle inhibitor protein with the main function to slow down or even stop the cell division cycle [49]. Nitti et al., 2002 [50] showed that low p27^Kip1^ protein expression in gastric adenocarcinoma relates to advanced tumours, it is significantly higher in weakly differentiated cases and is perceived as a negative prognostic agent for patient’s survival. Zheng et al., 2005 [51] first reported that p27^Kip1^ expression, cell cycle arrest, and apoptosis are strongly correlated in the SGC7901 cell line. Overexpression of p27^KIP1^ was associated with the induction of apoptosis and the extension of cell cycle in G1 phase of SGC7901 cells.

p21^Cip1^, also known as cyclin-dependent kinase (CDK) inhibitor 1, which can inhibit all cyclin/CDK complexes and it is a main target of p53 operations [52]. p21 protein expression progressive decreases from normal gastric mucosa, chronic superficial gastritis, and precancerous gastric lesions to gastric cancer was reported by Luo et al., 2014 [53]. They also suggested that the loss of p21 expression was very closely correlated with stadium of tumour differentiation, area of invasion, vascular invasion, Lauren classification, and metastasis to lymph node. It seems that p21 occurs in a role of tumour suppressor in the development and expansion of gastric cancer.

## 7. Factors that Regulate Apoptosis Process

Many studies have been investigated on *p53* genetic alterations in gastric carcinomas. It became clear that *p53* mutational changes show up in early stages of cancer development and their frequency is much higher with the cancer development process [54]. *P53* mutations are detected in 0–77% of gastric cancer cases [55] and they are significantly more frequent in proximal lesions than in distal. They mostly occur in exons 4–11, and they have several hot spots at codons 175, 245, 248, 273, 282, and 213. G:C > A:T transitions at CpG sites and are therefore are very popular types of alterations in GC patients [56]. P53 immunoreactivity is observed in 17–90.7% of invasive gastric cancer cases. Nuclear staining of p53 is not similar between diffuse and intestinal gastric carcinomas, with distinctly higher appearance in intestinal types [57]. Molecular analysis of gastric cancer that was performed by Cristescu et al., 2015 [58] showed the different gastric cancer subtypes are assigned to the different clinical outcomes and the TP53 is also a part of division of molecular gastric cancer. The group revealed that the tumor protein 53 active and TP53-inactive subtypes incorporate patients with intermediate prognosis and recurrence rates, however TP53-active group displays more promising prognosis.

The cell surface receptor programmed death-1 (PD1) and its ligand (PDL1) have been described in gastric tumours as markers assigned to poor prognosis of the patient [59]. Screening of clinical importance of PD1 and its ligands’ expression, as well as T cell infiltration might be a future perspective for using this as biomarkers in GC immunotherapy [60]. Correlation with clinical features showed that PDL1 membranous expression was in around 38% of tumour cells among the analysed cases, as well as 75% of infiltrating immune cells. The expression of PDL1 was visibly higher in a group of patients without metastasis, with PCNA and C-met expression, as well as *EBV* positive group. The study that was conducted by Böger et al., 2016 [61] displayed that PD-L1 expression was importantly dominated among men with GC, *Her2/neu* positive, *Epstein Barr* virus positive, microsatellite instable, *PIK3CA*-mutated, GCs of the proximal stomach, unclassified, and papillary. The significantly better patient’s outcome was correlated with the high PD-L1/PD-1 expression was associated with a significantly better patient outcome. The connection of PD-L1/PD-1 expression with various clinico-pathological characteristics might supply as a surrogate marker of PD-L1-positive gastric carcinomas, as well as can allow for improving the immune checkpoint treatment possibilities [61].

The *p73* gene encodes for a protein with high similarity to this displayed by *p53*. Kang et al., 2000 [62] described the expression of p73 in gastric carcinoma tissues. Importantly, low levels of p73 expression were detected in noncancerous gastric tissues and analysed cell lines, whereas p73 was detected in 94.9% of carcinoma tissues. The study demonstrated that p73 is not an aim of genetic modifications in gastric carcinogenesis. Wild-type p73 is very often overexpressed in gastric cancer tissues by the activation of a silent allele or transcriptional induction of an active allele.

Murine double minute gene 2 (*mdm2*) is a newly described oncogene that is placed at chromosome 12q13-14 [63]. Studies on the expression of mdm2 revealed that it is increasing in gastric cancer [64]. Moreover, expression level of the MDM2 protein is significantly higher in intestinal metaplasia in comparison to chronic gastritis [65]. Subtypes of intestinal metaplasia show different patterns of expression of the mdm2 and p53 protein [66]. Their expression is increased in atypical intestinal metaplasia (AIM) and gastric carcinomas in comparison to simple intestinal metaplasia (SIM). AIM may display the precancerous nature of gastric carcinoma more largely than SIM or the conventional IM subtypes. Moreover, AIM may be concerned as a preneoplastic lesion and be a significant indicator in the clinical follow-up of GC patients.

B-cell lymphoma 2 (Bcl-2) expression and increased risk of gastric carcinoma recurrence was studied by Wu et al., 2014 [67]. There is still a lack of predictive indicators for GC recurrence, therefore, the determination of such factors, like it was performed for bcl-2 expression, might be applicable after curative resection in patients with gastric cancer. In fact, the researchers displayed that peritoneal recurrence occurred as the mostly observed type after curative gastrectomy. Moreover Wu et al., 2014 [67] showed that lymph node metastases, depth of invasion, as well as negative expression of bcl-2 were correlated with a higher probability of recurrence action.

## 8. Multidrug Resistance Related Proteins

DNA repair protein complementing XP-A cells is a protein that in humans is encoded by the *XPA* gene [68]. *XPA* A23G and *XPC* exon 8 Val499Ala polymorphisms were explored to be effective markers for the recognition of individuals at risk of developing gastric cardiac adenocarcinoma (GCA) [69]. D’Errico et al., 2006 [70] have displayed that mutation in the *XPC* gene are responsible for accumulation of oxidative damage in human cells. ‘A’ allele of *XPA*-23G > A has been correlated with increased levels of places tender to formamidopyrimidine DNA glycosylase that shows oxidative DNA damage in the lymphocytes of carriers [71]. Polymorphisms in both genes can impact on destroying the capability to look for bulky adducts and oxidative DNA damage. This might promote the accumulation of DNA lesions, and therefore increased gastric cancer risk.

Expression of multidrug resistance-associated protein 2 (MRP2) was detected in several carcinomas, which was reported by Sandusky et al., 2002 [72]. High expression of MRP2 is important in the in initial absence of reaction to chemotherapy treatment of tumor, and therefore, it might be a significant biomarker for chemotherapy response. Immunohistochemistry was conducted on zinc formalin-fixed tissue. Immunostaining was detected in neoplastic cells on the cell membrane with M2-lll-6 antibody against MRP2 and cell membrane and cytoplasm with EAG5 against MRP2. Among examined cancers MPR2 was localized in eight of 13 gastric carcinomas and the expression was increased in well differentiated tumours. MRP2 is associated with irinotecan and anthracyclines transport, therefore the clinical applications of pharmacogenetics might allow for accurately defining the appropriate drug and dose for individual treatment [73].

The multidrug resistance 1 gene (*MDR1*) is very important candidate gene in developing gastric cancer susceptibility [74]. A total 365 gastric cancer patients and 367 controls were genotyped using the designed restriction site-polymerase chain reaction method to look for single genetic polymorphisms (SNPs) of the *MDR1* gene. The outcome showed the allele and genotype frequencies of c.159G > T and c.1564A > T to be statistically different among both analysed groups in the Chinese Han population. Detected variants could play a role as molecular markers in early gastric cancer diagnosis. Zhu et al., 2013 [75] reported that MDR1 has a significant impact on drug resistance answer, and the knockdown of MDR1 might reverse this phenotype among gastric cancer cells. To present the exact role of MDR1, the group performed knockdown of MDR1 expression using shRNA in gastric cancer cells that are resistant to drugs. The results were reviewed to assess Adriamycin (ADR) accumulation and drug sensitivity. Using two shRNAs it was possible to inhibit the expression of mRNA and protein of MDR1 in SGC7901-MDR1 cells. In final observation the group concluded *MDR1* knockdown to cause decreasing of ADR collection in cells and increment of susceptibility to ADR treatment.

The expression of Glutathione S-transferases Pi (GST-P), also known as GST-π, is readily higher in tumours chemically induced. In response to the tumor creation, GST-π is expressed as a resistance mechanism by which cells can survive [76]. The expression of GST-π in patients with gastric carcinoma was increased in comparison to normal mucosa (51.3% versus 23.2%), which was reported by Yu et al., 2014 [77]. The correlation of GST-π expression was observed with sex (male versus female, 59.7% versus 35.7%, *p* < 0.05) and differentiation (well, moderately, and poorly, 40.5%, 41.9%, and 64.7%, respectively, *p* <0.05). Moreover, high expression of GST-π was connected to the tumor invasion, recurrence, as well as poor prognosis.

## 9. Mucins with Impact on Cell Membrane Properties

Mucins are a family of extracellular large molecular weight, heavily glycosylated proteins (glycoconjugates). Both membrane bound mucins and secreted mucin possess many shared features [78]. Their important properties have related to capability to form gels, with main functions regarding lubrication, cell signalling, and the creation of chemical barriers. Mucins are frequently engaged in an inhibitory role [79]. Overexpression of mucin proteins, MUC1, MUC2, MUC5AC, and MUC6 have been reported in to be present in the gastric carcinogenesis process [80]. The correlation between the expression of several mucins and clinicopathologic profiles and patient’s survival are presented in Table 1.

## 10. Factors that Influence High Progression of Gastric Cancer and Peritoneal Metastasis

**Figure 1:**
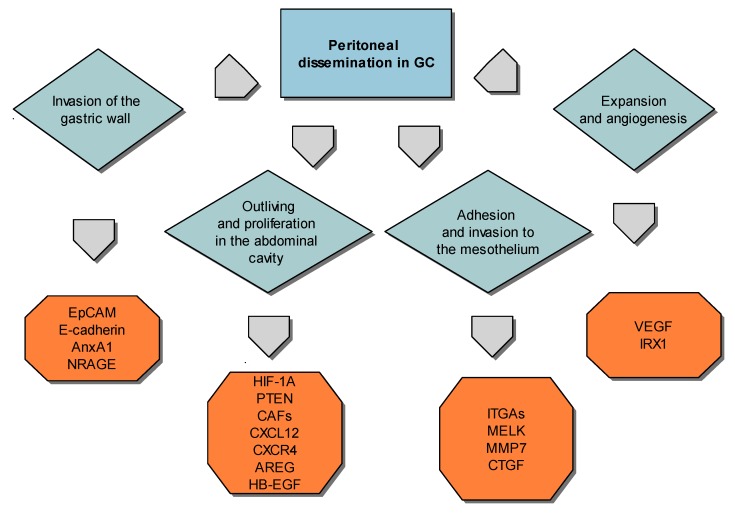
Peritoneal dissemination steps and molecular markers.

In metastatic gastric cancer, expansion into the peritoneal cavity is present in more than 55–60% of patients [85]. Peritoneal dissemination is an important clinical indication, resulting in poor prognosis [86]. Recently, Cristescu et al., 2015 [58] investigated a gene expression dataset of 300 primary gastric tumors to sectionalize four molecular subtypes of GC, which were connected with different patterns of molecular alteration, prognosis of GC patients, progression, and cancer prognosis, which encompass MSS/TP53^+^ subtype, MSS/TP53^−^ subtype, MSI subtype, and MSS/EMT subtype. The MSS/EMT subtype includes diffuse tumors type of GC with very poor prognosis, with the disposition to come up in young age and the highest recurrence frequency (63%) of the four classified subtypes is present [58]. An insightful view of the molecular occurrence of each step of peritoneal dissemination is shown in Figure 1, indicating markers of prognosis that are described in the next four sections. Described molecules may contribute to the multiple steps; we tried to highlight their impact on each process while taking into consideration their putative primary involvement to peritoneal dissemination.

### 10.1. Gastric Cancer Cells Invasiveness

Epithelial cellular adhesion molecule (EpCAM) is a factor that is involved in physical homophilic interaction between intestinal epithelial cells (IECs) and intraepithelial lymphocytes (IELs) at the mucosal epithelium, and it is involved in first line of defence against mucosal infection [87]. The EpCAM expression has been investigated in GC patients with peritoneal metastasis (PM) [88]. They reported that the expression of EpCAM was visibly higher in the PM lesions when compared to the primary lesions. Therefore, the intraperitoneally dosed EpCAM antibody may possibly play a role to contribute to the anti-cancer outcome in PM lesions of GC. In conclusion, the authors gave the suggestion that only GC cells with an increased level of EpCAM have a potential to metastasize to the peritoneum.

E-cadherin (epithelial-cadherin), which was encoded by the *CDH1* gene, is a transmembrane glycoprotein that takes part mainly in cell-cell adhesion maintenance. Its role is regarded to the signalling pathways, which controls cell proliferation, migration, survival, and invasion [89]. Dysregulation of E-cadherin allows for gastric carcinoma development by disturbing gastric epithelial cells [90]. E-cadherin is a tumor suppressor that has been described to be downregulated in gastric cancer [22]. They highlighted the abnormal E-cadherin expression and some connections with tumor stage, grade, depth of invasion, and therefore it might be useful as a predictor for tumor invasiveness in gastric cancer patients. Moreover, genetic alterations in the *CDH1* gene and epigenetic factors, such as DNA hypermethylation, are significant factors to decrease E-cadherin in GC development [91]. The authors observed high correlation of promoter hypermethylation of *CDH1* with gastric cancer, which shows that epigenetics and *CDH1* are important to GC ethology. The group also mentioned that genetic variant C-160A in *CDH1* was associated with cardia, intestinal and diffuse GC. The association between *H. pylori* infection and promoter methylation of *CDH1* gene were studied by Perri et al., 2007 [92] and *CDH1* methylation was categorized as an early event in *H. pylori* gastritis. The environmental effect of *H. pylori* infection on methylation status was assigned to the *H. pylori* eradication therapy in reversal methylation profile among patients with chronic gastritis [93]. In 1998, the linkage between germline mutation in *CDH1* gene and the genetic cause of hereditary diffuse GC (HDGC) was revealed [94]. Germline *CDH1* mutations are connected to the increased lifetime risk of occurring diffuse gastric (DGC). Intestinal-type GC cases do not belong to the HDGC type, and among these families there is no evidence to analyze the *CDH1* mutation [95].

Annexin A1 (AnxA1) is well described glucocorticoid-regulated anti-inflammatory protein. The exogenous and endogenous form of annexin A1 counter-regulate the actions of inborn immune cells, specifically extravasation and the formation of proinflammatory mediators, which control the appropriate level of activation to be reached but not overstepped [96]. The role of AnxA1 in GC survival was described by Cheng et al., 2012 [97]. High expression of AnxA1 was correlated with peritoneal metastasis and serosal invasion. Additionally, AnxA1 expression was certainly associated with invasiveness of human gastric cancer cells both in vitro and in vivo studies. The regulation of cell invasion by AnxA1 was also observed in this study, by the formyl peptide receptor (FPR)/extracellular signal-regulated kinase/integrin beta-1-binding protein pathway, in which all three FPRs (FPR1-FPR3) were committed in this regulation process.

Neurotrophin receptor-interacting melanoma antigen-encoding gene homolog (NRAGE) induces cell apoptosis and suppresses cell metastasis [98]. To understand the role and the clinical importance of NRAGE in GC, Kanda et al., 2016 [99] reported the expression levels of *NRAGE* and its putative interacting genes. Their studies showed the correlation between a higher level of *NRAGE* mRNA expression in GC patients in comparison to normal tissues. Both NRAGE mRNA and protein levels were strictly correlated. Increased NRAGE expression in GCs was connected to the disease-free survival and described as an autonomous marker for GC prognosis. Additionally, expression of *NRAGE* mRNA was observed to be correlated with that of apoptosis antagonizing transcription factor *(AATF*), as well as *NRAGE* knockdown importantly reduced the proliferation, migration, and invasion actions of GC cells.

### 10.2. Expansion of Peritoneal Dissemination

Peritoneal dissemination very often shows in late-stage GC and it is perceived as a crucial problem that shortens the survival time of patients with gastric carcinoma [100]. Peritoneal milky spots (PMSs) are omentum-associated lymphoid tissues that have been described as the area of origin of immature PMS macrophages [101]. The hypoxic microenvironment is engaged in supervising the tumor stem cell phenotype and is connected to the patient’s prognosis through hypoxia-inducible factor-1α (HIF-1α), which is a major transcriptional factor that corresponds to hypoxic stimuli. While the peritoneal dissemination is expanding, the gastric cancer stem/progenitor cells (GCSPCs) are eligible to come into PMSs, in which the hypoxia is maintained. Miao et al., 2014 [102] displayed the correlation of the higher expression of HIF-1α and gastric cancer peritoneal dissemination (GCPD) among GC cases. The GCSPC population expanded in primary gastric cancer cells when hypoxia was present in vitro, also hypoxic GCSPCs displayed intensified self-renewal action, but decreased differentiation ability, which was interceded by HIF-1α. The authors concluded the results to PMSs role, which is assigned to serve a hypoxic niche and favour GCSPCs peritoneal dissemination through HIF-1α.

The tumor suppressor gene phosphatase and tensin homolog (PTEN) is one of the major factors in decreasing tumor growth, expansion, and metastasis [103]. Zhang et al., 2014 [104] revealed a unique action by which PTEN can prohibit the growth and invasion of gastric carcinoma. This mechanism is regarding the downregulation of focal adhesion kinase (FAK) expression and highlights that exploiting *PTEN/PI3K/NF-κB/FAK* axis is an auspicious direction to treat gastric cancer metastasis. The results of this investigation showed that the high expression of PTEN or knockdown in GC cells in consequence provoked the downregulation or the upregulation of FAK, and lowered or raised cell invasion, appropriately. Additionally, FAK increased expression might redeem the inhibition of cell invasion by PTEN. PTEN can inhibit PI3K/NF-κB pathway and reduce the DNA binding of NF-κB on the FAK promoter. Ma et al., 2017 [105] have been reported that low expression of PTEN was detected in a large part of GC tissues, which displayed important associations with differentiation grade in GC patients. PTEN knockdown encouraged the expansion and invasion of cells and could lead to an expected increase in p-AKT, p-GSK-3β, β-catenin, E-cadherin, MMP-7, MMP-2, and MMP-9 in GC cells.

Cancer-associated fibroblasts (CAFs) are apparently responsible for the invasion process and metastatic actions in some cancers, among them gastric cancer is titled, through the activation of CXCL12/CXCR4 signalling [106]. The group investigated increased CXCL12 expression levels, which correlated with grater tumor size, higher tumor depth, lymphatic invasion, and poor prognosis in GC. Stimulation CXCL12/CXCR4 by CAFs interceded integrin β1 clustering at the cell surface increases invasiveness of GC cells. Yasumoto et al., 2006 [107] visibly showed the CXCR4/CXC12 axis actions in the development of peritoneal carcinomatosis from gastric carcinoma. They conducted the calculation of *CXCR4* mRNA expression levels among NUGC4 cells, and it was increased, also the cells displayed sprightly migratory actions, answering to its ligand CXCL12. The CXCL12 intensified proliferation and fast escalations in the phosphorylation of protein kinase B/Akt and extracellular signal-regulated kinase of NUGC4 cells. Analysis showed that 67% primary tumours of the stomach with peritoneal metastasis were positive for CXCR4 expression, in comparison to 25% with another distant metastasis were positive. Explicitly, 85% of CXCR4-expressing primary tumours raised peritoneal metastases. CXCR4 positivity of primary gastric carcinomas meaningfully was associated with the evolution of peritoneal carcinomatosis.

Yasumoto et al., 2011 [108] were interested in whether epidermal growth factor receptor (EGFR) ligands play any role in the expansion of peritoneal spread from gastric cancer. According to their observations, an increased concentration of the EGFR ligands amphiregulin, heparin-binding EGF-like growth factor (HB-EGF) and CXCL12, occurred in malignant ascites. High expression levels of EGFR and CXCR4 mRNA and protein were observed among primary tumours and human gastric cancer cell lines, with an upraised possibility to evoke peritoneal carcinomatosis. Amphiregulin AREG and HB-EGF were provoking factors of intensified proliferation, migration, and functional CXCR4 expression in gastric cancer NUGC4 cells, with the overexpression of CXCR4. These observations drew attention to the EGFR ligands amphiregulin and HB-EGF as important factors, interacting with the CXCL12/CXCR4 axis, in the development of peritoneal spread from gastric cancer.

### 10.3. Adhesion of Gastric Carcinoma Cells to the Peritoneum

The connection between peritoneal lining and GC cells is a major move in peritoneal dissemination. Takatsuki H et al., 2004 [109] showed that, the peritoneal implantation of NUGC-4 human gastric carcinoma cells in athymic mice, dosing of the cells with anti-alpha2 or anti-alpha3 integrin antibody decreased the number of disseminated nodules. Moreover, suppression by the anti-alpha3 integrin antibody was more powerful than that by the anti-α2 integrin antibody. The cDNAs to human α2 and α3 integrins (ITGAs) were applied into K562 leukemic cells. Those cells displayed a positive occurrence of the integrin beta1 subunit and negative of the α2 or α3 subunit. The α3 integrin-transfected cells were adhered to the monolayer of peritoneal mesothelial cells intensely in comparison to others. Reverse transcription-PCR was applied to detect the expression of laminin-5 and laminin-10/11, which were both high-affinity ligands for alpha3beta1 integrin. mRNA for these isoforms was observed in mesothelial cells from the diaphragm and parietal peritoneum. To conclude, α3β1 integrin is important factor in activation the first steps in attachment of cancer cells to the peritoneum, and therefore allowing for the creation of peritoneal metastasis.

Maternal embryonic leucine zipper kinase (MELK) is a serine/threonine-protein kinase that is engaged in multiple processes, including cell cycle regulation, self-renewal of stem cells, apoptosis and splicing regulation. It has a significant impact on cell proliferation and carcinogenesis [110]. Du et al., 2014 [111] described the expression of mRNA and protein level of MELK in gastric cancer. Reduction the proliferation, migration, and invasion actions of GC cells were observed after the knockdown of MELK activity, as well as lower number of cells in the G1/G0 phase and higher those in the G2/M and S phases. Further knocking down of the MELK expression was assigned to the decreased quantity of actin stress fibers and inhibited RhoA operations. At the end, the scientists reported that the knockdown of MELK reduced the phosphorylation of the FAK and paxillin, and prevented gastrin-stimulated FAK/paxillin phosphorylation. To summarize the MELK importance, it is an essential factor for causing cell migration and invasion via the FAK/Paxillin pathway, and therefore, it is crucial in GC development.

Matrix metalloproteinase-7 (MMP-7) is an enzyme that plays a role in matrix degrading and impacts on the occurring of invasion and metastasis in gastric cancer patients [112]. Yonemura et al., 2000 [113] investigated experiments to check the machinery creation of peritoneal dissemination among GC cases. The MMP-7 protein level and mRNA expression were studied in primary gastric cancers and peritoneal dissemination. The obtained results revealed the overexpression of *MMP-7* mRNA in 53% of cases regarding primary gastric tumours, whereas in the normal gastric mucosa, fibroblasts, and mesothelial cells, there was negative expression of this marker. Immunohistochemistry analysis displayed the MMP-7 immunoreactivity on the cell membrane and the cytoplasm of GC cancerous cells. The higher MMP-7 protein level was observed among 53% of primary cancers and MMP-7 tissue status had a statistically relevant correlation with lymph node metastasis, weak differentiation, and peritoneal dissemination. Examined GC cases with MMP-7 expression had a worse survival rate and an increased risk of death of peritoneal recurrence. The 100% of investigated peritoneal disseminations expressed *MMP-7* mRNA and 93% revealed immunoreactivity to anti-human MMP-7 monoclonal antibody. The results suggested the substantial role of MMP-7 in the creation of peritoneal dissemination in gastric cancer.

Connective tissue growth factor (CTGF) has been reported to be involved in peritoneal metastasis and gastric cancer progression [114]. High expression of CTGF, as well as treatment with the recombinant protein considerably reduced cell adhesion [115]. CTGF enduring transfectants displayed a lower number and size of tumor nodules in the mesentery, during in vivo peritoneal metastasis. Overexpression of CTGF among GC patients was significantly correlated with the earlier TNM staging and an increased survival rate after the surgery. Coimmunoprecipitation analysis showed that CTGF binds to integrin α3. These results demonstrated that the mediation of GC peritoneal metastasis is occurring through integrin α3β1 attaching to laminin, and CTGF efficiently stops the cooperation by binding to integrin α3β1.

### 10.4. Peritoneal Spreading and Neovascularization

High expression of *IRX1* gene, which codes iroquois-class homeodomain protein IRX1, was strongly connected with the growth arrest in GC cases. Additionally, the overexpression of *IRX1* gene suppresses peritoneal expansion and spread the tumor metastasis [116]. Human umbilical vein endothelial cells (HUVECs) and chick embryo and SGC-7901 gastric cancer cells were operated for angiogenesis and vasculogenic mimicry (VM) tests. For detecting the function of the downstream target gene of *IRX1*, the bradykinin receptor B2 (*BDKRB2*), small interfering RNA was applied. Results showed the suppression of peritoneal spreading with the reduction of angiogenesis as well as VM creation. The supernatant from SGC-7901/*IRX1* cells, revealed a strong inhibiting effect on angiogenesis both in vitro and in chick embryo.Reduction of tube formation, cell proliferation, and migration, as well as invasion in vitro, were reported by gene-specific RNA interference for *BDKRB2*, or its effector *PAK1*, which codes serine/threonine-protein kinase PAK1. It is now proved that enforcing *IRX1* expression, importantly suppresses peritoneal expansion and pulmonary metastasis via anti-angiogenesis and anti-VM mechanisms.

The important role of angiogenesis in cancer and the cloning of vascular endothelial growth factor (VEGF) in 1989 focused the research studies on this issue and allowed for the important clinical translation of VEGF—directed therapies to the clinic [117]. VEGFR2 receptor expression is limited to vasculature and it is a very significant factor in angiogenesis. Its activation in consequences allow for actuation a complex cascade of downstream signalling pathways that are caused by VEGF receptors, which further results in neovascularization, vasodilation, higher vascular permeability, and migration of bone marrow endothelial cells [118]. VEGF blockade inhibits these pathways and effects tumor survival, migration, and invasion. Advanced gastric cancer is still a huge challenge to find an effective treatment. Monoclonal antibody Ramucirumab can block VEGFR2 activation by binding to it. Randomized phase III trial, called REGARD, was using ramucirumab treatment in comparison to the placebo for patients with advanced, pre-treated gastric cancer, which achieves its initial endpoint of increased total survival [119]. Another RAINBOW trial of paclitaxel with ramucirumab versus paclitaxel with placebo for advanced pre-treated gastric cancer approved the increased survival benefit of ramucirumab, as an antiangiogenic cure among GC patients. Therefore, ramucirumab is the first FDA approved treatment for advanced gastric cancer after previously applied chemotherapy. Recently Roviello et al., 2017 [120] performed a meta-analysis to check the efficacy and the safety of the novel VEGFR-2 inhibitors among cases with metastatic gastric and gastroesophageal junction cancer. A meta-analysis that is based on the literature of randomized controlled trials (RCTs) was investigated. A significant impact of overall survival, which was increased, was detected. This study confirmed the anti-VEGFR-2 inhibitors, like apatinib and ramucirumab positive action [120].

Cyclooxygenase-2 (COX-2) expression is connected to the angiogenesis and *Helicobacter pylori* infection among gastric cancer patients [121]. Immunohistochemical stain against cyclooxygenase 1 and 2 has been investigated among samples that were resected from patients with GC. Among the 72 analysed patients, cases with cyclooxygenase 2-positive staining were tightly correlated with vascular invasion and *H. pylori* infection, as well as poorer prognosis. On the other hand, multivariate analysis displayed that vascular invasion, serosal invasion, and lymph node metastasis were autonomous prognostic factors for patients with gastric cancer, but cyclooxygenase 2 expression was not.

Knowing the apoptotic and proliferative levels among gastric cancer patients may be helpful to find better treatment options and prevention of developing GC. The importance of Ki-67, caspase-3, and p53 expression levels were demonstrated by Xiao et al., 2013 [122], and were correlated with clinical data among patients with GC. The Ki-67 and p53 expression was correlated with tumor-node-metastasis staging. Detection of increased caspase-3 and p53 expression were assigned to the intestinal-type of GC. Correlation between three tested agents: Ki-67, caspase-3, and p53 was statistically significant. Positive correlation among caspase-3 expression and adverse prognosis of GC patients was displayed by the Kaplan-Meier analysis. Additionally, the Cox’s proportional hazards model was used to show that several clinical data, such as: age, gender, depth of invasion, lymphatic invasion, lymph node metastasis, TNM staging, Lauren’s classification, and caspase-3 expression were autonomous prognostic agents for gastric cancer patients. In conclusion, expression of Ki-67, caspase-3, and p53 can be perceived as factors that influence the differentiation and progression of GC.

## 11. Conclusions

Gastric cancer is a highly heterogeneous disease. Advantageous studies of the molecular biomarkers of gastric cancer have been deeply investigated, in outcome provided a wide spectrum of signatures in this area. This review summarized the recognition patterns of gastric cancer, including cell cycle regulators, factors that regulates apoptosis, microsatellite instability, multidrug resistance proteins, factors that influence cell membrane properties, module of HER2 expression, and agents with impact on the progression of gastric cancer and peritoneal metastasis. Novel investigation must be done to display specific signatures for the early diagnosis of gastric cancer and for the prediction of chemo/radiotherapy. Nowadays, is it possible to apply high throughput technologies, such as whole genome and exome sequencing, to find various biomarkers with prognostic and diagnosis potential. It is a promising tool to obtain auspicious outcomes to further clinical applications.

## Figures and Tables

**Table 1:** Mucins and their impact on patient’s survival with gastric cancer.

MUC Type	Function	Expression Patterns in Gastric Cancer	Authors
MUC1	Protective role by binding to pathogens, functions in a cell signalling capacity, presented in the nucleus regulation the activity of transcription factor complexes, which take part in tumor-induced changes of host immunity system	MUC1 positive cases highly overexpressed in intestinal-type carcinomas, increased rate of vascular invasion and lymph node metastasis, lower 5-year survival rate	Wang et al., 2016 [81]
MUC2	The major intestinal mucin, expressed by goblet cells of the small intestine and colon, important role in organizing the intestinal mucus layers at the epithelial surface; forming trimers that crosslink with TFF3 and Fcγbp, allow to highly viscous extracellular layer	Strongly correlated with the intestinal histological type, the correlation between MUC2 and HER2 expression is possible to demonstrate the connection between the intestinal differentiation of cancer cells and HER2 expression	Park et al., 2015 [82]
MUC5AC	Glycoprotein of gastric and respiratory tract epithelium guards the mucosa from infection and chemical damage, thanks to binding to inhaled microorganisms and particles, which are later removed by the mucociliary system	Decreased Muc5AC expression was importantly correlated with poor overall survival, additionally, decreased Muc5AC expression was also meaningfully reported to the tumour invasion depth and lymph node metastasis	Zhang et al., 2015 [83]
MUC6	Might deliver a mechanism for modulation of the composition of the protective mucus layer connected to acid secretion or the presence of bacteria and noxious agents in the lumen, involvement in the cytoprotection of epithelial surfaces	MUC6 is a marker of gastric foveolar cells and antral/cardiac mucous glandular cells, reflect to gastric phenotypes	Kim et al., 2013 [84]
